# Supplementary material for: Development and evaluation of AI model with deep learning for segmentation of extraocular muscles in thyroid eye disease
Source: PLoS One. 2026 May 26;21(5):e0349074. doi: 10.1371/journal.pone.0349074 (PMC13210272; doi:10.1371/journal.pone.0349074)

Supplimental Material (S1 Appendix)

S1 Appendix: Five-fold cross-validation used in the current study.

The input data for the model was divided equally into five folds.(model 1 to 5) The evaluation scores for all folds were averaged to determine the final evaluation score.


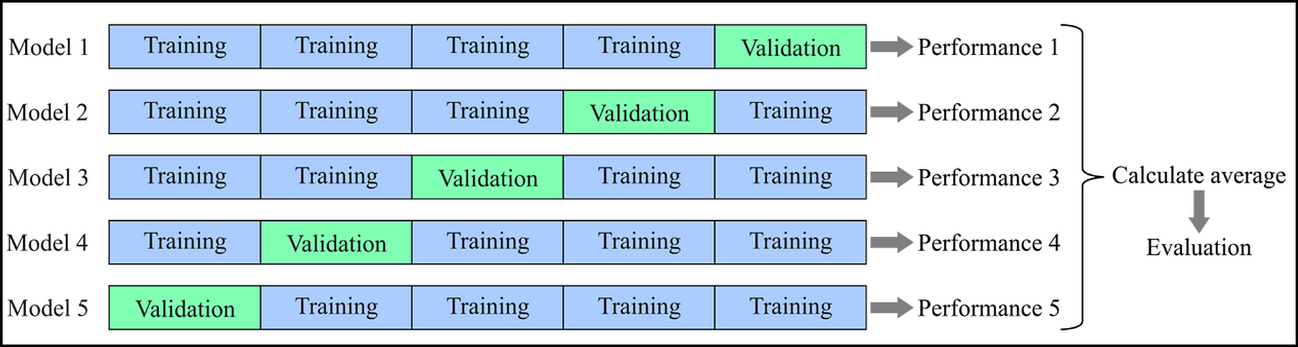

Supplement: S1 Appendix — The input data for the model was divided equally into five folds.(model 1–5) The evaluation scores for all folds were averaged to determine the final evaluation score. (DOCX) [file pone.0349074.s001.docx]
